# The Impact of Individual and Context-Related Factors on Students’ Reactions After Sexual Assault: A Vignette Study

**DOI:** 10.1177/08862605251319008

**Published:** 2025-02-23

**Authors:** Irena Bošković, Melissa de Roos, Leonie Maatz, Robin Orthey

**Affiliations:** 1 Erasmus University Rotterdam, the Netherlands; 2 Aoyama Gakuin University, Tokyo, Japan

**Keywords:** sexual assault, students, post-assault reactions, factors

## Abstract

University students are often victims of sexual assault (SA) with a wide range of severity, but they are the least likely to disclose the assault or to take any concrete (legal) steps against the perpetrator. Prior work reported 13 main factors that influence university students’ choice of reaction post-assault: (a) Fear of personal consequences, (b) distrust in authorities, (c) downplay of assault severity, (d) psychological factors, (e) situational factors, (f) lack of evidence, (g) emotional factors, (h) fear of interpersonal consequences, (i) social factors, (j) giving a benefit of doubt, (k) seeking justice, (l) needing support, and (m) presence of witnesses. In this experimental study, we included a student sample pre-screened not to have a history of SA (*N* = 419), and we provided them with a vignette. Vignettes were either neutral (control condition, *n* = 32) or manipulated to present each of listed factors (13 conditions, 26 < *n_s_* > 33). Students were randomly assigned to 1 of 14 conditions in total and were asked to imagine being a protagonist who was assaulted and to rate the likelihood of 8 different post-SA reactions (tell friends, tell family, confront the person, report, police report, do nothing, try to forget, and [falsely] deny). We investigated to see which of the 13 factors had the most impact on each of the reactions. Overall, our results indicate that, when comparing the manipulation groups to the neutral condition, social factors (e.g., religious family, stigma) have the highest impact on students’ decision-making post-assault. Social factors increase the likelihood of all passive reactions (e.g., false denial, *contrast* = 1.82, *p* < .001) and decrease the odds of taking pro-active actions (e.g., making the report, *contrast* = −0.96, *p* = .002). The implications and the limitations of this study are discussed.

Sexual assault (SA) is one of the most traumatic experiences, with detrimental effects on both physical and mental health, as well as on interpersonal adjustment (Eisenberg et al., 2021). The National Institute of Justice (2010) categorizes SA as a wide range of unwanted sexual behaviours that are completed against a person’s will, without their consent, or without the victim’s ability to consent, and may involve actual or threatened physical force, coercion, intimidation, or pressure. This could include, for example, sexual touching, voyeurism, exhibitionism, and undesired exposure to pornography, but does not include penetration. Other definitions also include non-consensual threatened, attempted, or completed oral, anal, or vaginal penetration with body parts or objects, which constitutes rape (Krebs et al., 2016; Morgan & Kena, 2018; National Institute of Justice, 2010). Yet, defining what constitutes SA is a difficult task, given that official definitions vary internationally due to different legislations and individual perceptions(European Union Agency for Fundamental Rights, 2014).

Although SA occurs cross-culturally and across different genders and age groups, victims are most commonly young and female (Conley et al., 2017; Dworkin et al., 2021). For instance, college-aged women (i.e., 18–24 years old) experience higher rates of victimization than any other age groups (Conley et al., 2017; Sinozich & Langton, 2014). Still, these results mostly reflect the prevalence of assault among students in the United States. The prevalence and characteristics of SA among European university students have only recently started gaining the attention of researchers (Boskovic et al., 2023; Blanco et al., 2022; Hagerlid et al., 2023; Rudolfsson et al., 2024; but see also European Union Agency for Fundamental Rights, 2014). It has been shown that more than half of European university students have experienced assaults of varying severity, with the most frequent incidents involving *groping*. The most vulnerable victims were female students and students who identify as members of the LGBTQIA+ community (Boskovic et al., 2023), which aligns well with findings from other countries (e.g., United States; see Cantor et al., 2020; Conley et al., 2017; Dworkin et al., 2021; Eisenberg et al., 2017; Krebs et al., 2016; Sinozich & Langton, 2014; and also the UN Women report, 2021). Despite a high frequency of SA among European students, the majority do *not* disclose the assault in ways that would result in legal consequences for the perpetrator (Boskovic et al., 2023). Specifically, after the assault, students most commonly choose to first tell their friends about it (>30%), do nothing (>25%), or confront the person directly (~20%). Students rarely report the perpetrator (<7%), either to the police or to a person in charge, and some students also (falsely) deny the assault when asked about it (3%–5%; Boskovic et al., 2023). Thus, assaulted students mostly seek support from their friends but do not take further actions against the perpetrator (see also Fisher et al., 2003; Moore & Baker, 2018; National Union of Students, 2019; Orchowski & Gidycz, 2012; Orchowski et al., 2009; Sabina & Ho, 2014).

In order to increase the level of assault disclosure and reporting, it is of high importance to discover specific circumstances or factors that determine students’ reactions post-assault (e.g., Ahrens et al., 2007; Khan et al., 2018; Moore & Baker, 2018). A higher level of disclosure and reporting has been shown to improve survivors’ post-assault adjustment (Jaffe et al., 2022; Orchowski et al., 2013); therefore, a better understanding of influential factors could directly help build better support and guidance for SA victims. Findings obtained from both U.K.- and U.S.-based students showed that the most common obstacles to reporting the assault were its high emotional burden on the survivor (shame, embarrassment), psychological barriers (e.g., low confidence in one’s memory), fear of retaliation, situational factors surrounding the assault (e.g., alcohol intoxication at the time), as well as fear of stigma and interpersonal consequences (see Ahrens et al., 2007; Bureau of Justice Statistics, 2014; Khan et al., 2018; Lorenz & Ullman, 2016; National Union of Students, 2010, 2019; Orchowski & Gidycz, 2012). Therefore, there is a plethora of reasons why students opt to conceal the assault.

The above-mentioned findings were replicated in a recently conducted study among students from three European universities by Boskovic et al. (2023). In this study, students with a history of SA were asked to describe the situation they were in, their actions post-assault, and the reasoning behind these actions. Researchers closely inspected the narratives and grouped the most commonly reported reasons behind students’ decision-making post-assault into 13 impactful factors, such as: (a) fear of personal consequences (e.g., losing a job), (b) distrust in authorities and others’ actions (e.g., doubt they would be believed), (c) low severity assumptions (e.g., “just groping”), (d) psychological factors (e.g., memory distrust), (e) situational factors (e.g., alcohol), (f) lack of evidence (e.g., no proof), (g) emotional factors (e.g., shame), (h) fear of interpersonal consequences (e.g., changes in friends group dynamics), (i) social factors (e.g., religion, stigma), and (j) giving the benefit of the doubt (e.g., protecting the perpetrator). These factors, as shown previously, mostly supported *not* taking actions against the perpetrator (see Hagerlid et al., 2023; Sabina & Ho, 2014). In contrast, three other factors motivated students to take action: (k) seeking justice, (l) needing support, and (m) the presence of witnesses (Boskovic et al., 2023). Overall, these results show that, in the aftermath of an assault, it is equally important to understand individual and contextual factors both separately and in interaction.

## Current Study

In this project, we experimentally tested the impact of the above-listed 13 factors using vignettes depicting the most commonly reported type of assault among university students—groping (of a female student) in public by a male stranger (Boskovic et al., 2023). Our participants were students who, following ethical restrictions, did not have a history of SA. They were provided with 1 of 14 possible versions of the vignette. In total, there were 14 different vignettes (conditions): 1 neutral and the descriptions of each of the 13 factors (e.g., lack of witnesses and fear of interpersonal consequences; see Materials and Questionnaire section). Students were randomly allocated to one of the conditions (between-subjects design). The students’ task was to read the vignette, imagine themselves as the protagonist, and then rate the likelihood of different reactions post-assault. The goal was to test which factor, compared to the neutral condition, significantly impacts students’ decision-making post-assault and in what direction (passive or active approach). This was the first experimental attempt to address these questions, which differed significantly from prior research on the topic (e.g., by using hypothetical situations and employing a pre-screened sample to exclude individuals with prior SA experience). Despite these differences, we still expected to replicate the key finding from Boskovic et al. (2023), which suggested that conditions such as seeking justice, having support, and the presence of evidence would provide higher likelihood ratings for action-oriented reactions (e.g., reporting) and lower ratings for passive reactions (e.g., doing nothing) than the control condition.

## Method

### Participants

Based on a power calculation using G*power (Faul et al., 2009), with an alpha of .05 and beta of .95, 154 participants were needed for the study, including all 14 conditions. However, to ensure appropriate statistical analyses, the desired number of participants increased to 280, with *n* *=* 20 per condition.

The participants were recruited in two different ways, from our university participant pool (*N* = 461) and a broader pool of students recruited via social media (*N* = 253). Due to ethical restrictions, only subjects without a history of an assault were allowed to proceed with participation, which led to the withdrawal of 25.3% of students from the first sample (*n* = 117), and 34.7% from the second one (*n* = 88). Further, we only included students, so 37 non-students recruited via social networks were removed. We also specifically asked our participants to allow us to use their data and 14 participants did not provide permission (11 from the internal and 3 from the external sample). Finally, incomplete answers (*n* = 38) and participants who reported low English proficiency (*n* = 1; see Procedure) were also removed.

This resulted in a total of 325 students of our university (i.e., internal sample) and 94 external students, leading to the final sample of 419 participants (aged 17–44, *M* *=* 21.50, *SD* *=* 2.91). The majority of the sample (84.2%, *n* *=* 353) was female, 15.0% (*n* *=* 63) were male, two participants (0.5%) were nonbinary, and one person stated their gender as “other.” Overall, participants rated their English proficiency as good to excellent (*M* *=* 4.28, *SD* *=* 0.66, range 3–5), with no significant difference between students from internal and external samples [*t* (417) = −1.34, *p* = .180]. The samples, however, did differ in age [*t* (417) = −9.45, *p* < .001], with the internal sample having an average age of 20.85 years (*SD* *=* 2.66, range 18–44) and the external sample being significantly older on average (*M* = 23.78 years, *SD* *=* 2.60, range 17–37). However, looking between students allocated to different conditions, there was no significant difference with regard to age [*F*(13, 405) = 0.40, *p* *=* .970] nor English proficiency [*F*(13, 405) = 1.16, *p* *=* .306].

This study was approved by the standing ethical committee of Erasmus University Rotterdam, Erasmus School of Social and Behavioural Sciences, Rotterdam, the Netherlands.

### Materials and Questionnaire

#### Vignettes

In order to test the impact of 13 factors previously reported (Boskovic et al., 2023) as impactful on students’ reactions post-assault, we randomly allocated our participants to 1 of 14 groups, 13 depicting one of the factors and a control (neutral) condition. In each condition, participants were given a vignette depicting the same situation but in different circumstances (e.g., lack of evidence and fear of personal consequences). Participants were asked to read the vignette and imagine being the protagonist in the given situation. Below are all 14 vignettes. A part of the vignette presented in italic was identical across all conditions, and we only present the original text added in each group.

##### Control Condition

*Sasha came to a club with a group of friends. Sasha went alone to the bar to order a drink and is about to pick it up when s/he suddenly feels that someone is strongly and persistently groping her/his behind.* The person even shoves their hand between Sasha’s legs. Sasha is thinking about what to do. Now, please take a moment and imagine being Sasha.

##### Lack of Evidence

The person even shoves their hand between Sasha’s legs. Unfortunately, no one else saw what happened, and Sasha cannot identify anyone. There is no evidence of what happened, so Sasha is thinking about what to do. Now, please take a moment and imagine being Sasha.

##### Fear of Personal Consequences

The person even shoves their hand between Sasha’s legs. Sasha turns around and realizes the person groping her/him is the boss from her/his student job. If Sasha does something, s/he might lose the job, so Sasha is thinking about what to do. Now, please take a moment and imagine being Sasha.

##### Fear of Interpersonal Consequences

The person even shoves their hand between Sasha’s legs. Sasha turns around and sees that the person that groped her/him is a friend of her/his best friend. Sasha knows that it is likely that her/his reaction will reflect on the relationship with her/his best friend. Sasha is thinking about what to do. Now, please take a moment and imagine being Sasha.

##### Distrust in Authorities and Their Actions

The person even shoves their hand between Sasha’s legs. “If I told someone about what just happened, would they even believe me?,” s/he thinks. Based on her/his experiences, Sasha feels as though s/he cannot trust authorities because in the past, Sasha was never taken seriously when s/he reported incidents that s/he experienced or witnessed. Sasha is thinking about what to do. Now, please take a moment and imagine being Sasha.

##### Situational Factors

The person even shoves their hand between Sasha’s legs. Sasha already had a few drinks before and really does not want to make a scene, as s/he is out with friends tonight. Sasha is thinking about what to do. Now, please take a moment and imagine being Sasha.

##### Social Factors

The person even shoves their hand between Sasha’s legs. Sasha comes from a background where groping or molesting is never openly discussed and is often overlooked or even justified as a “compliment” instead of sexual molestation. Also, Sasha’s parents are highly religious and judgmental about any kind of inappropriate contact until Sasha is married. Sasha is thinking about what to do. Now, please take a moment and imagine being Sasha.

##### Psychological Factors

The person even shoves their hand between Sasha’s legs. Sasha does not really understand what just happened and goes back to her/his friends. After a couple of hours, it suddenly hits her/him that what had happened was not appropriate. Still, Sasha has doubts about whether the act actually happened as s/he now remembers it or whether it was different. Sasha is thinking about what to do. Now, please take a moment and imagine being Sasha.

##### Seeking Justice

The person even shoves their hand between Sasha’s legs. S/he becomes annoyed and angry, and s/he wants the person groping her/him to be embarrassed or punished so that her/him and others will be protected from this person. Sasha is thinking about what to do. Now, please take a moment and imagine being Sasha.

##### Low Severity Assumption

The person even shoves their hand between Sasha’s legs. S/he is not okay with that, but s/he thinks that s/he is at a bar, so people touching each other is not uncommon. Also, it’s not like someone raped her/him. Sasha is thinking about what to do. Now, please take a moment and imagine being Sasha.

##### Emotional Factors

The person even shoves their hand between Sasha’s legs. Sasha feels shocked and very ashamed. Sasha thinks that maybe s/he provoked it somehow. Sasha is confused and is thinking about what to do. Now, please take a moment and imagine being Sasha.

##### Needing Support

The person even shoves their hand between Sasha’s legs. Sasha is the type of person that seeks support in any situation in which s/he is not sure how to act. Besides, her/his closest friends are lawyers. Sasha is thinking about what to do. Now, please take a moment and imagine being Sasha.

##### Giving a Benefit of Doubt

The person even shoves their hand between Sasha’s legs. Sasha then notices that the person standing next to her/him starts hugging the person who groped her/him. Sasha thinks that maybe the person who groped her/him had mistaken her/him for their friend. Still, Sasha is thinking about what to do. Now, please take a moment and imagine being Sasha.

##### Presence of Evidence and Witness

The person even shoves their hand between Sasha’s legs. When Sasha looks to her/his side, s/he sees someone signaling her/him that they see what had happened. They come over to Sasha and say, “I have everything on video.” Sasha is thinking about what to do. Now, please take a moment and imagine being Sasha.

#### Likelihood of Reactions

After reading the vignette, participants were asked to indicate the likelihood of taking the listed reactions on a 5-point Likert scale ranging from 1 (*very unlikely*) to 5 (*very likely*).

The list of reactions included: Telling friends, telling family, doing nothing, confronting the person, denying anything happened, reporting to a person in charge, reporting to the police, and trying to forget. Participants were also given an option to add a reaction of their own and report its likelihood as well.

### Procedure

The study was conducted using Qualtrics. After providing an informed consent, participants were asked about their own history of SA. This was done to ensure that we followed ethical requirements for the study, specifically, that participation in our project would not inflict unnecessary additional trauma and to avoid confounding. Participants were not explicitly asked whether they experienced SA. Instead, they were asked whether anyone had ever committed or attempted sexual actions toward them without the participant’s consent or when they were unable to consent. The reasoning behind such an approach is that many victims of SA would not label this experience as such (Kahn et al., 2003; Lonsway & Archambault, 2012). If they responded affirmatively, they were provided with contact information for support options and forwarded to the end of the survey. If participants reported not having any direct assault experience, or if they only knew someone who had it but did not experience it themselves, they were forwarded to further questions (i.e., demographic questions). We also asked our participants to rate their English proficiency using a 5-point scale (1 = “terrible” to 5 = “excellent”). To answer the central question of this study, participants were forwarded to a randomly assigned vignette condition describing a SA and were asked to imagine themselves as the protagonist of the story. Afterward, participants were asked to imagine themselves in the depicted situation and to indicate the likelihood of taking each presented reaction (5-point Likert scale). Additionally, they were given the possibility to add another reaction they deemed suitable. After the task, participants were presented with exit questions regarding motivation, discomfort, difficulty, and clarity (rated on a 5-point scale with higher values indicating higher levels) and were asked to indicate whether they had previously experienced a situation as described in the vignette (i.e., whether they had experienced a mild SA/groping). Lastly, participants were informed about the purpose of the study and supplied with resources for psychological support if needed.

### Data Analyses

The 13 experimental conditions were compared with the control condition on the variables of motivation, discomfort, difficulty, and clarity using multivariate analysis of variance and multivariate analyses of covariance (MANCOVA). Significant main effects were followed up with multiple comparisons using Bonferroni correction. Each experimental group was compared to the control group across all eight reactions using simple contrasts. Data is available at Open Science Framework platform: https://osf.io/6jpwy/.

## Results

### Motivation, Discomfort, Difficulty, and Clarity

Overall, our participants reported not only very high motivation to participate in our study (*M* = 4.31, *SD* = 0.76; range: 2–5) but also moderate discomfort (*M* = 3.03, *SD* = 1.07, range: 1–5). Students reported moderate difficulty (*M* = 3.62, *SD* = 1.02, range: 1–5) and high clarity of the task they were asked to complete (*M* = 4.76, *SD* = 0.56, range: 1–5). There was no significant effect of condition (i.e., the presented vignette) on participants’ motivation, discomfort, perceived difficulty, and clarity [λ = .898, *F* (52, 1559.05) = 0.95, *p* *=* .772, η*p*² = .03].

### Likelihood Ratings of Post-Assault Reactions

For participants in the neutral, *control condition*, telling friends was rated as the most likely reaction (*M* *=* 4.78, *SD* *=* 0.42), followed by confronting the person (*M* *=* 3.97, *SD* *=* 1.15) and trying to forget (*M* *=* 3.28, *SD* *=* 1.17). In contrast, denying that anything happened (*M* *=* 1.53, *SD* *=* 0.80), doing nothing (*M* = 1.94, *SD* = 0.98), and police reporting (*M* = 2.22, *SD* = 1.29) were rated as least likely.

We then compared the likelihood ratings of each reaction between the control condition and the manipulated factor. Before conducting the main analysis, a Mann-Whitney *U* test was performed to evaluate whether the likelihood ratings of eight reactions differ between the internal and external sample. It was found that the students of our university, when compared to the external sample, had significantly higher ratings for the reactions of doing nothing (*U* = 12080.00, *z* *=* −3.22, *p* *=* .001, *r* = −.16), confronting the person (*U* = 12678.50, *z* *=* −2.66, *p* *=* .008, *r* *=* −0.13), falsely denying (*U* *=* 1267.50, *z* *=* −2.71, *p* *=* .007, *r* *=* −.13), and trying to forget (*U* *=* 13192.50, *z* *=* −2.09, *p* *=* .036, *r* *=* −.10). Therefore, for these reactions, a MANCOVA was conducted while controlling the sample (internal vs. external) (Table 1).

**Table 1.** Likelihood Ratings of Reactions Post-Assault Across Conditions.

Reactions	Condition/Factors (*M, SD*)													
	Control	Lack of Evidence	Fear Personal Consequences	Fear Interpersonal Consequences	Distrust in Authorities	Situational Factors	Social Factors	Psychological Factors	Seeking Justice	Low Severity	Emotion Factors	Needing Support	Benefit of Doubt	Presence of Evidence
*N*	32	31	27	31	32	31	26	27	30	33	30	27	30	32
Telling friends	4.78 (0.42)	4.68 (0.48)	4.71 (0.47)	4.06 (1.15)	4.56 (0.84)	4.55 (0.81)	3.35 (1.09)	4.67 (0.48)	4.77 (0.43)	4.36 (0.93)	4.67 (0.84)	4.63 (0.74)	4.57 (0.73)	4.56 (0.76)
Telling family	2.38 (1.10)	2.52 (1.36)	2.79 (1.42)	2.39 (1.20)	2.81 (1.18)	2.45 (1.41)	1.54 (0.76)	2.70 (1.46)	3.33 (1.24)	2.36 (1.30)	2.53 (1.14)	2.89 (1.19)	2.10 (1.06)	3.13 (1.36)
Do nothing	1.94 (0.98)	2.58 (1.12)	1.89 (0.97)	2.23 (1.23)	2.28 (1.33)	2.06 (1.09)	3.69 (1.19)	2.59 (1.25)	1.83 (0.79)	2.55 (1.44)	2.17 (1.09)	2.04 (0.98)	2.33 (1.18)	2.00 (1.02)
Confronting the person	3.97 (1.15)	3.94 (1.03)	3.63 (0.93)	4.00 (0.89)	4.22 (0.94)	3.77 (0.99)	2.23 (1.21)	3.44 (1.09)	4.23 (0.86)	3.70 (1.13)	4.07 (0.91)	3.96 (1.10)	3.07 (1.34)	3.84 (1.05)
Denial	1.53 (0.80)	1.97 (1.17)	1.89 (0.85)	2.00 (1.13)	1.78 (0.91)	1.81 (0.95)	3.39 (1.10)	1.96 (0.98)	1.47 (0.68)	2.03 (1.33)	1.67 (0.92)	1.63 (0.70)	1.80 (0.96)	1.47 (0.84)
Report to a person in charge	3.19 (1.28)	3.19 (1.08)	2.85 (1.10)	2.26 (1.03)	3.34 (1.31)	3.16 (1.39)	2.23 (1.11)	2.74 (1.35)	4.00 (0.70)	2.94 (1.27)	3.07 (1.17)	3.04 (1.16)	2.30 (1.02)	4.06 (1.08)
Police report	2.22 (1.29)	2.06 (1.00)	2.11 (1.19)	1.74 (1.13)	2.19 (1.06)	2.10 (1.11)	1.62 (0.75)	1.96 (0.98)	2.83 (1.15)	2.09 (1.16)	2.23 (1.04)	2.63 (1.28)	1.53 (0.73)	2.91 (1.35)
Trying to forget	3.28 (1.17)	3.52 (1.09)	3.48 (1.22)	3.58 (0.99)	3.66 (1.04)	3.23 (1.36)	4.58 (0.86)	3.52 (1.16)	2.87 (1.11)	3.73 (0.98)	3.30 (1.26)	2.93 (1.27)	3.33 (1.21)	3.16 (1.25)

In order to gain the clearest overview, we used simple contrasts and compared each of the 13 conditions to the neutral one for every reaction (*p* levels were adjusted for the number of comparisons, see Table 2). We found that the likelihood of *telling friends* about the incident diminishes when there is a presence of fear of interpersonal consequences [*t*(61) = −3.72, *p* < .001, *d* *=* −0.84] and social factors [*t*(56) = −7.11, *p* *=* <.001, *d* *=* −1.80]. *Telling family* was more likely with a factor such as seeking justice [*t*(60) = 3.04, *p* *=* .003, *d* *=* 0.80]. Further, respondents were more prone to *doing nothing* when social factors [*t*(56) = 5.73, *p* < .001, *d* *=* 1.62] were present.

**Table 2.** Simple Contrasts of Likelihood Ratings of Reactions Post-Assault (1–5) of the Experimental Conditions Compared to the Control Group.

Reactions	Mean Differences (Control—Experimental Condition; *p*-Value)													
	Control	Lack of Evidence	Fear Personal Consequences	Fear Interpersonal Consequences	Distrust in Authorities	Situational Factors	Social Factors	Psychological Factors	Seeking Justice	Low Severity	Emotion Factors	Needing Support	Benefit of Doubt	Presence of Evidence
*N*	32	31	27	31	32	31	26	27	30	33	30	27	30	32
Telling Friends	4.78	−0.10 (.590)	−0.08 (.698)	−**0.72 (<.001)**	−0.22 (.253)	−0.23 (.227)	−**1.44 (<.001)**	−0.12 (.566)	−0.02 (.940)	−0.42 (.028)	−0.12 (.555)	−0.15 (.448)	−0.22 (.270)	−0.22 (.253)
Telling family	2.38	0.14 (.652)	0.40 (.215)	0.01 (.969)	0.44 (.160)	0.08 (.807)	−0.84 (.011)	0.33 (.312)	**0.96 (.003)**	−0.01 (.971)	0.16 (.616)	0.51 (.114)	−0.28 (.384)	0.75 (.016)
Do nothing^a^	1.94	0.65 (.023)	−0.08 (.796)	0.28 (.325)	0.33 (.239)	0.14 (.611)	**1.70 (<.001)**	0.64 (.029)	−0.10 (.733)	0.59 (.034)	0.22 (.436)	0.09 (.764)	0.38 (.189)	0.05 (.860)
Confronting the person^a^	3.97	−0.04 (.892)	−0.32 (.242)	0.04 (.883)	0.26 (.319)	−0.21 (.430)	−**1.70 (<.001)**	−0.52 (.059)	0.26 (.327)	−0.26 (.315)	0.10 (.697)	0.00 (.992)	−**0.89 (<.001)**	−0.12 (.658)
Denial^a^	1.53	0.44 (.072)	0.34 (.179)	0.46 (.058)	0.24 (.318)	0.29 (.239)	**1.82 (<.001)**	0.42 (.093)	−0.06 (.807)	0.49 (.042)	0.13 (.594)	0.09 (.720)	0.26 (.300)	−0.07 (.767)
Report to a person in charge	3.19	0.01 (.984)	−0.34 (.269)	−**0.93 (.002)**	0.16 (.590)	−0.03 (.929)	−**0.96 (.002)**	−0.45 (.141)	0.81 (.006)	−0.25 (.389)	−0.12 (.682)	−0.15 (.620)	−**0.89 (.003)**	**0.88 (.003)**
Police report	2.22	−0.15 (.580)	−0.11 (.709)	−0.48 (.087)	−0.03 (.910)	−0.12 (.661)	−0.60 (.039)	−0.26 (.367)	0.62 (.029)	−0.13 (.641)	0.02 (.959)	0.41 (.155)	−0.69 (.015)	0.69 (.013)
Trying to forget^a^	3.28	0.24 (.413)	0.19 (.535)	0.29 (.309)	0.37 (.199)	−0.05 (.872)	**1.27 (<.001)**	0.23 (.440)	−0.41 (.159)	0.44 (.124)	0.02 (.959)	−0.36 (.228)	0.04 (.888)	−0.13 (.645)

*Note.* Significant contrasts are presented in bold.

a The respective reactions were analyzed controlling for sample as a covariate; *p* levels adjusted with the new critical value being .004 (.05/13 = .004).

In alignment with that, the likelihood ratings for *confronting the assaulter* were significantly diminished when social factors [*t*(56) = −6.16, *p* < .001, *d* *=* −1.48), and a benefit of doubt [*t*(60) = −3.35, *p* *<* .001, *d* *=* −0.72) were present. *Falsely denying* the assault was significantly more likely if social factors were present [*t*(56) = 7.12, *p* < .001, *d* *=* 1.97). Choosing to *report to a person in charge* was less likely if there was a fear of interpersonal consequences [*t*(61) = −3.18, *p* *=* .002, *d* *=* −0.81), social factors [*t*(56) = −3.12, *p* *=* .002, *d* *=* 0.80), or giving the perpetrator the benefit of doubt [*t*(60) = −3.01, *p* *=* .003, *d* *=* −0.77). Yet, the presence of evidence [*t*(62) = 3.02, *p* *=* .003, *d* *=* 0.74) had an opposite, positive effect. *Going to police* was not significantly impacted by any of the factors, which could be due to the perceived “low-severity” nature of the depicted assault (groping). Lastly, the odds of *trying to forget* were increased by social factors [*t*(56) = 4.18, *p* < .001, *d* *=* 1.25], whereas the other factors did not significantly change the ratings when compared to the control group.

### Other Possible Reactions

In addition to the predetermined reactions, participants were also given the opportunity to state another reaction with an accompanying likelihood. However, as these reactions differed a lot in their nature and likelihood ratings, they could not be meaningfully compared and analyzed. Overall, 85 participants chose to add an additional reaction. Possible reactions included, for example, becoming angry or aggressive toward the person, talking to a psychologist about the event, and leaving the venue/going home.

### Personal Assault Experience Post-Manipulation Check

At the end of the study, we asked whether our participants had ever previously experienced the situation as described in the vignette (i.e., groping). Across all conditions, 23.6% of participants indicated having experienced a situation as described in the vignette, although those same participants denied being assaulted at the beginning of the study. When asked to elaborate, students mostly stated that they did not think that the situation constituted SA or that they expected the present study to be about “more serious assault,” and that they were not sure about the incident or could not identify a perpetrator. These statements align with the most commonly reported barriers to reporting SA.

In order to test whether the history of SA did have an impact on participants’ responses, we repeated the main analyses using only the *control* group (with SA history *n* = 11 vs. without SA history *n* = 21), but we found no significant differences on any of the reactions (*F*s < 1.65; *p*s > .21; *U*s < 114.5, *p*s > .123).^1^

### Additional Check: Gender

We examined whether the likelihood ratings of the eight reactions differed by gender. First, we examined the control condition, which included 5 male and 27 female participants. A marginal difference was observed for reporting to a person in charge (*U* = 30.00, *z* = −0.332, *p* = .053; *d* = 0.73), with males providing higher ratings (*M* = 4.20, *SD* = 1.30) than females (*M* = 3.00, *SD* = 1.21). Next, we combined all conditions and analyzed gender differences in likelihood ratings for all reactions (*n*_males_ = 63, *n*_females_ = 353). We again found significant differences in ratings of reporting it to a person in charge (*M*_males_ = 3.41, *SD*_males_ = 1.31, and *M*_females_ = 2.97, *SD*_females_ = 1.15; *U* = 8958.50, *z* = 2.56. *p* = .010; *d* = 1.25), but also in police reporting (*M*_males_ = 2.70, *SD*_males_ = 1.30, and *M*_females_ = 2.07, *SD*_females_ = 1.10; *U* = 7965.00, *z* = 3.75. *p* = <.001, *d* = 1.13) and trying to forget (*M*_males_ = 2.98, *SD*_males_ = 1.44, and *M*_females_ = 3.52, *SD*_females_ = 1.12; *U* = 8831.50, *z* = 2.70. *p* = .007; *d* = 1.18). Overall, males rated action-oriented reactions as more likely than females (i.e., reporting), whereas females showed higher ratings for passive reactions (e.g., trying to forget).

## Discussion

In this vignette study, we experimentally tested which factors impact the likelihood of different reactions post-assault. Namely, some of the factors, such as fear of interpersonal consequences or having a religious family, were more individual, whereas some others, such as situational (e.g., alcohol consumption) were more reliant on the context in which the situation occurred. It is important to note that all of our participants declared not to have prior SA history before they participated in our study, hence, their ratings could not have been impacted by their prior experience but rather by the vignette they read.

Our findings can be summarized as follows: First, looking at the control condition, the most common reactions students opt for are to confide in their friends and/or confront the person directly. These results confirm prior findings indicating that students primarily seek support from their peers (see Boskovic et al., 2023; Fisher et al., 2003; Moore & Baker, 2018). Students provided the lowest likelihoods for denying the assault, doing nothing, and police reporting albeit these findings do not align with previous results obtained on students with SA history.

Specifically, although false denial was shown to be a reaction of a small minority (3%–5%) of students in prior work, similar to police reporting (<7%), doing nothing was shown to be the most common reaction among students post-assault (Boskovic et al., 2023; Orchowski et al., 2013; National Union of Students, 2019). This discrepancy in the likelihood ratings of doing nothing could be explained by the selection of our sample. Specifically, we included only students who declared *not* to have any SA history at the beginning of the study.

This selection was not only due to the ethical restrictions because of the possibility of re-traumatizing our participants, but also in order to control the effect our vignettes have on the ratings without the impact of students’ prior experience. This is important to note, as it was shown that students without a history of SA may be less accurate when estimating their reactions post-assault. Specifically, Boskovic et al. (2023) inspected this issue and showed that students without actual SA history underestimate the frequency of passive responding to the assault and overestimate reporting behaviour post-assault when compared to students with SA history.

Second, when looking at 13 specific conditions and comparing the likelihood ratings of different reactions to those in the control condition, each of the reactions, except police reporting, had a significant change under some specific set of factors. The lack of significant impact on the police reporting reaction is not surprising considering that the likelihood ratings for this reaction were relatively low across all of the conditions, which aligns with prior findings (e.g., Amnesty International Netherlands, 2021). Further, the depicted assault was pertaining to groping, which is often perceived by students as “not serious enough” to involve police (e.g., National Union of Students, 2010, 2019). In contrast, looking at the most likely reactions, it was shown that two individual factors significantly *diminish* the chances of *telling friends* about the assault—fear of interpersonal consequences and social factors. As telling friends involves good social adaptation and is based on a bond we make with others, it is unsurprising that potential negative impact on that social bond (e.g., the perpetrator being a friend of our friend) would lower the willingness to disclose the assault (Fisher et al., 2003). Similar logic can apply to the understanding of the negative impact of social factors. Namely, the social factor condition included additional information that suggested that Sasha comes from a religious and traditional family with a judgmental attitude toward sexual behaviour. Hence, telling others about the assault might be unlikely not only due to the attitude of our parents but more importantly due to the internalized stigma (Sabina & Ho, 2014; Sinko et al., 2021; Kennedy & Prock, 2018). However, our results show that the emotional factors, which included shame, for instance, did not impact the likelihood ratings differently than the control condition. Thus, in their decision-making, students might put more weight on how they are perceived by others rather than on how they are feeling.

*Confronting the person* directly is commonly reported as students’ post-assault reaction (Moore & Baker, 2018), yet, we showed that, similarly to above, social atmosphere can significantly lower the odds of engaging in this reaction, as well as giving the benefit of a doubt that the perpetrator did not mean to assault us. Interestingly, the next reaction, *reporting to a person in charge*, was negatively impacted by all three so far mentioned factors—fear of interpersonal consequences, social factors, and benefit of the doubt—diminishing the odds of this reaction. But the presence of evidence was also a significant influence, enhancing the likelihood of making such a report. This finding is aligned with prior studies (Boskovic et al., 2023) and our expectations, suggesting that students felt more secure in making a report once having evidence of an assault, which in our vignette condition was video footage.

The rest of the reactions mostly included *not* taking action, except telling family, which was rated on a similar level of likelihood by each of the conditions and by the control group. *Trying to forget* is a commonly reported coping strategy post-assault, especially among children who were assaulted (Goodman-Brown et al., 2003). The social factors (i.e., rigid and religious family) significantly enhanced the odds of opting for this reaction, once again confirming the negative effect a judgmental environment can have on post-assault reactions. Yet, interestingly, social factors did not significantly impact the disclosure of the assault to family members (i.e., *telling family* reaction). Instead, seeking justice was the only significant factor supporting this reaction, partially confirming our hypothesis. This result indicates that individual traits, such as relying on fairness, can overcarry the (potentially negative) effect the environment might have on our decision-making (see Moore & Baker, 2018; see also Orchowski et al., 2013). Our findings also showed that none of the examined 13 factors could potentially diminish the likelihood of taking the passive approach by *doing nothing*. This finding is not surprising as doing nothing was shown to be the most common response among students who experienced SA (Boskovic et al., 2023; Sabina & Ho, 2014). However, social factors, when compared to the control condition, were once again shown to have a significantly higher (supporting) impact on victims’ passive post-assault reaction. A very similar result was also found for *denial* as a reaction. Namely, both lack of reaction (i.e., doing nothing) and (false) denial of the assault present the underreporting behaviour, and our results confirm that closed-off environment, judgmental, and even religious family significantly increase the odds of SA survivors opting for it. Finally, our analyses of gender differences revealed that males exhibit higher likelihoods of action-taking and lower odds of remaining passive post-assault compared to females. This finding aligns with prior literature suggesting that males may be more likely to take action against the perpetrator (Boskovic et al., 2023). However, the use of hypothetical scenarios in this study limits the predictive value of these findings.

It is necessary to point out some *limitations* of this work. Namely, our sample was screened *not* to have a history of SA, which could limit the reliability of their answers as prior work showed that they are not accurate in predicting the reactions of students who experienced an assault (Boskovic et al., 2023). However, looking at the pre-screening exclusion number, we see that approximately a quarter of students had a history of SA, confirming a high prevalence of SA among students. Further, at the end of the study, we checked if students ever experienced a situation as such in a vignette, and again a quarter of students responded affirmatively. This finding confirmed the high prevalence of SA and highlighted that students often lack a clear understanding of what constitutes assault (Lichty & Gowen, 2021), with some not even recognizing actions such as someone reaching into their pants as assault (Boskovic et al., 2023). Although we checked if the history of SA had an impact on the control group’s likelihood ratings and found no significant differences, due to the low sample size, we cannot claim that prior history of SA did not impact students’ perception of depicted situations nor their likelihood ratings of post-assault reactions. Further, the sample size, although enough for the initial testing, should be larger, therefore capable of delivering more reliable results. Also, even though our analyses revealed certain gender differences in reactions’ likelihood ratings, the gender inequality within our sample was high; thus, the generalizability of our findings is limited, and further investigation is needed. Additionally﻿, the vignettes were possibly too brief for students to properly immerse themselves in the depicted situation and really imagine themselves as Sasha. Future studies could potentially include virtual reality, therefore increasing the ecological validity of responses students without SA history would provide. Finally, it is important to acknowledge that our study only included our university located in Western Europe, and that this type of research is necessary to conduct using diverse samples across different countries.

Despite the above-mentioned study limitations, our results are important in understanding the impact of a variety of factors on university students’ reactions to the most commonly reported type of assault they are exposed to (see Boskovic et al., 2023). Looking at the impact of each individual factor on the likelihood ratings of different reactions, it was found that fear of interpersonal consequences, social factors, seeking justice, benefit of doubt, and presence of evidence evoked significantly different ratings than those found in the control condition. From all these influential factors, when compared with the control condition, it seems that a rigid and judgmental close environment (i.e., social factors) poses the most *general* threat as it had a significant negative impact across different disclosure-related reactions, and it significantly increased likelihood ratings of reactions tying to forget and falsely denying the assault (.80 < Cohen’s *d_s_* < 1.97). This finding shows that the anticipated reactions from those close to them have an important influence on students’ decision-making post-assault. Thus, university policies and programs, created with the purpose of education and open discussion of SA (see also Holland, 2020), should engage not only their students but also those close to them, such as their families and friends. Further, from a criminal justice perspective, it is of relevance to highlight the results (i.e., effect sizes, despite insignificant adjusted p levels) that suggest the impact the presence/lack of evidence has on victims’ behaviour. Namely, while its presence increases the odds of the police report, its absence seems to be crucial in the victim’s decision to do nothing. Still, because these findings come from an experimental study with certain exclusion criteria, it needs to be noted that post-assault reactions might be significantly different if obtained from survivors of SA of various severity.

## Notes

1. We repeated the same analyses using the whole sample (with groups now being: With SA experience *n* = 99, and Without SA experience *n* = 320). Participants with prior SA provided significantly higher likelihood ratings than the rest of the sample for “telling friends” [*M*_sa_ = 4.68, *SD*_sa_ = 0.62 vs. *M* = 4.45, *SD* = 0.88; *F*(1,417) = 5.89, *p* = .02, η² = .014] and “doing nothing” [*M*_sa_ = 2.52, *SD* = 1.21 vs. *M* = 2.22, *SD* = 1.18; *F*(1,417) = 4.77, *p* = .03, η² = .011]. Yet, group with SA history provided significantly lower likelihoods than participants without prior SA experience on “telling family” [*M*_sa_ = 4.68, *SD* = .62 vs. *M* = 4.45, *SD* = 0.88; *F*(1,417) = 9.06, *p* = .003, η² = .021], “reporting to a person in charge” [*M*_sa_ = 2.78, *SD* = 1.25 vs. *M* = 3.13, *SD* = 1.26; *F*(1,417) = 5.77, *p* = .02, η² = .014], and “reporting to the police” [*M*_sa_ = 1.70, *SD* = 0.92 vs. *M* = 2.31, *SD* = 1.18; *F*(1,417) = 23.37, *p* < .001, η² = .05]. However, all participants provided similar ratings for the reactions “confronting the person” [*F*(1, 417) = .34, *p* = .56, η² = .001], “denying the assault” [*F*(1, 417) = .01, *p* = .91, η² = .000], and “trying to forget the assault” [*F*(1, 417) = 3.46, *p* = .064, η² = .008]. We repeated the same analyses using Kruskal-Wallis *H* test and replicated the same results.
